# Single-institutional outcome-analysis of low-dose stereotactic body radiation therapy (SBRT) of adrenal gland metastases

**DOI:** 10.1186/s12885-020-07030-w

**Published:** 2020-06-08

**Authors:** Theresa Voglhuber, Kerstin A. Kessel, Markus Oechsner, Marco M. E. Vogel, Jürgen E. Gschwend, Stephanie E. Combs

**Affiliations:** 1 Department of Radiation Oncology, Technical University of Munich (TUM), Ismaninger Straße 22, Munich, Germany; 2 Institute of Radiation Medicine (IRM), Helmholtz Zentrum München, Ingolstädter Landstraße 1, Neuherberg, Germany; 3 Deutsches Konsortium für Translationale Krebsforschung (DKTK), Partner Site Munich, Heidelberg, Germany; 4 Department of Urology, Technical University of Munich (TUM), Ismaninger Straße 22, Munich, Germany

**Keywords:** Stereotactic body radiation therapy, Adrenal gland metastases, Oncology, Outcome, SBRT, Toxicity, Survival

## Abstract

**Background:**

Adrenal gland metastases are a common diagnostic finding in various tumor diseases. Due to the increased use of imaging methods, they are diagnosed more frequently, especially in asymptomatic patients. SBRT has emerged as a new, alternative treatment option in the field of radiation oncology. In the past, it was often used for treating inoperable lung, liver, prostate, and brain tumors. Meanwhile, it is also an established keystone in the treatment of oligometastatic diseases. This retrospective study aims to evaluate the effect of low-dose SBRT in patients with adrenal metastases.

**Methods:**

We analyzed a group of 31 patients with 34 adrenal gland lesions treated with low-dose SBRT between July 2006 and July 2019. Treatment-planning was performed through contrast-enhanced CT, followed by image-guided stereotactic radiotherapy using cone-beam CT. The applied cumulative median dose was 35 Gy; the median single dose was 7 Gy. We focused on local control (LC), progression-free survival (PFS), overall survival (OS), as well as acute and late toxicity.

**Results:**

Seven adrenal gland metastases (20.6%) experienced local failure, 80.6% of the patients faced a distant progression. Fourteen patients were still alive. Median follow-up for all patients was 9.8 months and for patients alive 14.4 months. No treatment-related side-effects >grade 2 occurred. Of all, 48.4% suffered from acute gastrointestinal disorders; 32.3% reported acute fatigue, throbbing pain in the renal area, and mild adrenal insufficiency. Altogether, 19.4% of the patients faced late-toxicities, which were as follows: Grade 1: 12.9% gastrointestinal disorders, 3.2% fatigue, Grade 2: 9.7% fatigue, 6.5% headache, 3.2% loss of weight. The 1-year OS and probability of LF were 64 and 25.9%, respectively.

**Conclusion:**

Low-dose SBRT has proven as an effective and safe method with promising outcomes for treating adrenal metastases. There appeared no high-grade toxicities >grade 2, and 79.4% of treated metastases were progression-free. Thus, SBRT should be considered as a therapy option for adrenal metastases as an individual therapeutic concept in the interdisciplinary discussion as an alternative to surgical or systemic treatment.

## Background

The adrenal gland is a frequent target of malignant tumor cells of different entities, as it is characterized by a rich blood supply [1]. To make the right therapy decision, it is essential to differentiate between various benign and malignant neoplasia of the tissue. For example, pheochromocytomas arise from the cells of the adrenal medulla, while benign adenomas and metastatic lesions mainly form in the cortical regions [2]. Therefore, radiologic imaging is an indispensable tool for distinguishing between the most diverse types of tumors, as these have different characteristics on computed tomography (CT) and magnet resonance imaging (MRI) [3]. The diagnostic proceeding of adrenal metastases has undergone significant changes lately. In the past, lesions of the adrenal gland were incidental findings, manifested by symptomatic development, or were detected at autopsies [4, 5]. Since the introduction of systematic and more accurate tumor staging and the high-frequency use of imaging, the incidence of adrenal tumors has increased, as they are frequently discovered by chance, without any suggestive symptomatology [4, 6].

Once the adrenal gland metastasis is diagnosed through imaging procedures or biopsy, there are several options for curative and palliative treatment. Particularly for patients with isolated lesions, laparoscopic or open surgery is performed. Further treatment options are systemic chemo−/immunotherapy or ablative strategies such as cryoablation and radiofrequency ablation [3]. In the past, hypofractionated radiotherapy (RT) was often used for palliative purposes to reduce physical complaints caused by symptomatic adrenal metastases, such as back/flank pain, nausea, or hypoadrenalism [7]. With the introduction of stereotactic body radiation therapy (SBRT), a promising option for treating adrenal gland metastases appeared in the field of radiation oncology. Previously SBRT has been very extensively used in treating inoperable lung, liver, prostate, and brain tumors [8].

SBRT is characterized by its excellent local tumor control and is a good, non-invasive alternative treatment option, especially in patients, who decline any invasive- or ablative treatment, or when surgery is contraindicated due to multiple comorbidities [3, 6, 9–14]. The high-precision irradiation is applied in high single doses and few fractions. Due to the rapid dose-reduction outside the target volume, surrounding, healthy structures can be spared [6, 8, 15, 16]. This present retrospective study aims to contribute to the knowledge regarding hypofractionated low-dose SBRT of adrenal gland metastases. In particular, the primary endpoints local control (LC), progression-free survival (PFS) and overall survival (OS) will be evaluated as well as acute and late toxicity rates.

## Methods

### Patients

A group of 31 patients with a total of 34 irradiated lesions (3 patients had metastases treated on both adrenal glands) were evaluated. All were treated at the department of radiation oncology, Klinikum rechts der Isar, Technical University of Munich (TUM) between July 2006 and July 2019. Table 1 shows patient characteristics. The inclusion criterion was RT treatment with low-dose SBRT due to adrenal metastases. A progressive disease, uncontrolled primary and multifocal tumor manifestation were allowed. The guidelines of the german DEGRO (Deutsche Gesellschaft für Radioonkologie) working group for stereotactic RT (AG Stereotaxie) were applied to define SBRT. All patients were primarily treated in a palliative setting, for example, due to an oligoprogression in the area of the adrenal gland with otherwise stable tumor disease, to prolong survival, or to treat tumor dependent symptoms. An individual curative approach was attempted in three of the patients with oligometastases.

**Table 1:** Patient characteristics

Characteristics	Values
Number of patients (n)	31
Number of AGMs (n)	34
Gender	
Male	17 (54.8%)
Female	14 (45.2%)
Age at SBRT (median, range) [years]	66.1 (26.7–82.2)
Primary entities	
NSCLC	13 (41.9%)
Mamma Ca	6 (19.4%)
Melanoma	4 (12.9%)
Others	8 (25.8%)
Symptoms	
Present	2 (6.5%)
Absent	29 (93.5%)
KPS	
100%	1 (3.2%)
90%	19 (61.3%)
80%	10 (32.3%)
≤ 70%	4 (12.9%)
Location of AGMs	
Left	15 (48.4%)
Right	13 (41.9%)
Bilateral	3 (9.7%)
Controlled primary	
Yes	25 (80.6%)
No	6 (19.4%)
AGM diagnosis	
Synchronous	15 (48.4%)
Metachronous	19 (61.3%)
Metastases in other sites	
Yes	25 (80.6%)
No	6 (19.4%)
Systemic therapy within four weeks before/after SBRT	
Yes	19 (61.3%)
No	12 (38.7%)
FU-time (median, range) [months]	9.8 (0–120.5)

*AGM* adrenal gland metastasis, *SBRT* stereotactic body radiation therapy, *NSCLC* non-small-cell-lung-cancer, *KPS* Karnofsky Performing Score, *FU* follow-up

synchronous ≤3 months after initial primary diagnosis; metachronous > 3 months after initial primary diagnosis

Analysis of patient records and data collection took place after being approved by the local ethics committee of the Medical faculty of TUM, vote number 307/19.

### Treatment

Treatment planning was performed through contrast-enhanced CT. Five patients received an additional MRI and four patients an additional positron emission tomography-CT (PET-CT) for evaluating the tumor volume. Since 2010, 4D-CT was also acquired. Time-resolved imaging allows more accurate reconstruction of respiratory-related tumor movements, which also helps to protect surrounding healthy tissue [17, 18]. Before the 4D-CT was introduced, positioning the patient through frames, blue bag, abdominal press, wing board, and breathing techniques ensured motion management. Before irradiation, the patient’s exact location was controlled by using a cone-beam CT (CBCT) to ensure high-precision. During the planning simulation and during irradiation itself, patients were brought in a supine position and immobilized with the help of a vacuum mattress, a wingstep, and a knee wedge to further reduce irradiated uncertainties.

The median applied cumulative dose was 35 Gy (range: 25–57 Gy), and the median single dose was 7 Gy (range: 3–8 Gy) in 5 fractions (range: 3–14). This results in a median biological equivalent dose (BED) of 59.5 Gy (range: 37.5–72.0 Gy). The calculated doses were prescribed to the 60–80% isodose. Two patients received boost treatment with a total dose of 42 Gy (35 á 2.5 Gy base plan with a simultaneous integrated boost of 42 á 3 Gy) and 57 Gy (45 á 1.8 Gy base plan with a sequential boost of 12 á 4 Gy).

The treatment concept was determined in an interdisciplinary discussion, depending on tumor size, symptoms, and general condition of the patients.

### Follow-up

The first regular follow-up (FU) appointment was arranged 4–6 weeks after SBRT. Each additional exam took place approximately every three months in the first year and then every 6–12 months. Depending on tumor progression or worsening of symptoms, follow-up appointments could be individualized. Each appointment included a full physical exam, a consultation with a radiation specialist, and contrast-enhanced CT to assess the tumor status and therapy-associated side-effects. LC of the adrenal metastasis was assessed in each follow-up CT, regardless of the primary tumor status.

Every patient was examined for side-effects, such as fatigue, nausea, or abdominal pain, during and after treatment. We determined these side-effects according to the Common Terminology Criteria Adverse Events (CTCAE) Version 5.0. Due to the retrospective design of this study, the level of toxicity could not be precisely assessed in some cases; grading according to CTCAE was then carried out using documented follow-up reports, written by the supervising radio-oncologist and the development of the patient’s clinical course. We defined side-effects as either acute when they appeared within the first six months after RT or as late when they appeared after the six-months-period.

### Statistics

Based on the prevailing competing risks (e.g., death of a patient before a local failure occurred), the probability of LF was calculated using a competing-risk-analysis in order to increase accuracy [19, 20]. It was performed with R-Statistics (R-Foundation, Vienna). We used SPSS Statistics v25 (IBM, USA) for all other analyses.

For patients with multiple RT on both adrenal glands, the first treatment was used for the calculation of OS and PFS; for LC, all 34 cases were included. We calculated the LC from the last day of irradiation until local failure of the treated lesion or last day of follow-up. PFS was calculated from the last day of irradiation until general tumor progression or last day of follow-up; OS from the last day of irradiation until the date of death or the last date the patient was alive. Receiver operating characteristics (ROC) were used to determine thresholds for grouped variables. Cox regression was used for the analysis of subgroups. We considered a *p*-value of < 0.05 as significant.

The BED_10_ was calculated with the formula; BED (Gy) = dose/fraction x fraction number (1 + fraction dose / α/β); we used an α/β ratio of 10 for the tumor tissue, see Table 2 [21].

**Table 2:** Radiation parameters and treatment characteristics

ID	PTV (ml)	GTV (ml)	TD (Gy)	SD (Gy)	Fractions	Isodose	PTV-Dmax (Gy)	PTV-D2% (Gy)	PTV-D50% (Gy)	PTV-D98% (Gy)	GTV-D50% (Gy)	BED_**10**_
**1**	175.8	57.8	25.0	5.0	5.0	60%	42.8	42.3	37.7	29.0	41.3	37.5
**2**	376.0	225.5	25.0	5.0	5.0	60%	28.1	27.8	26.6	24.6	26.8	37.5
**3**	123.0	53.8	25.0	5.0	5.0	60%	41.8	41.7	39.6	29.7	41.1	37.5
**4**	45.0	x	25.0	5.0	5.0	60%	x	25.3	21.0	x	x	37.5
**5**	39.3	14.0	25.0	5.0	5.0	60%	42.0	41.5	34.5	26.3	39.1	37.5
**6**	37.3	8.3	35.0	7.0	5.0	65%	54.3	53.8	47.3	33.7	52.6	59.5
**7a**	40.7	12.9	40.0	8.0	5.0	65%	61.7	59.2	48.8	39.0	53.8	72.0
**7b**	24.1	6.9	40.0	8.0	5.0	65%	61.0	59.7	49.1	40.5	55.3	72.0
**8**	87.4	13.3	35.0	7.0	5.0	60%	47.1	46.3	40.4	22.8	44.5	59.5
**9**	71.9	21.0	25.0	5.0	5.0	60%	43.1	30.5	23.9	30.6	41.5	37.5
**10**	98.8	37.3	25.0	5.0	5.0	60%	x	25.5	23.3	x	24.6	37.5
**11**	69.8	25.6	40.0	8.0	5.0	65%	61.9	60.3	49.3	39.9	54.8	72.0
**12**	134.0	55.0	35.0	7.0	5.0	60%	x	37.7	31.9	x	34.0	59.5
**13**	122.2	51.3	35.0	7.0	5.0	60%	59.9	58.8	44.3	22.1	51.5	59.5
**14**	49.2	12.7	25.0	5.0	5.0	60%	41.9	41.9	38.6	30.1	41.0	37.5
**15**	82.8	33.0	25.0	5.0	5.0	80%	31.7	31.5	29.8	25.1	30.9	37.5
**16**	294.2	154.6	36.0	3.0	12.0	60%	62.7	59.2	47.6	33.3	50.6	46.8
**17**	434.8	247.5	27.0	5.4	5.0	60%	45.9	44.6	37.1	27.8	40.9	41.6
**18**	35.8	3.1	35.0	7.0	5.0	60%	61.8	60.8	47.6	31.9	58.9	59.5
**19**	81.6	22.3	35.0	7.0	5.0	60%	63.3	62.7	49.3	28.4	60.5	59.5
**20**	138.7	71.4	25.0	5.0	5.0	60%	39.5	37.7	26.3	25.3	27.6	37.5
**21**	58.5	12.2	35.0	7.0	5.0	60%	58.6	58.3	54.3	41.4	57.7	59.5
**22**	37.9	7.7	35.0	7.0	5.0	65%	56.0	54.8	43.2	24.9	50.7	59.5
**23a**	54.6	19.1	35.0	7.0	5.0	65%	55.9	55.3	46.7	35.8	53.2	59.5
**23b**	35.0	9.3	39.0	3.0	13.0	60%	68.2	67.0	50.3	36.4	62.7	50.7
**24**	25.1	4.2	36.0	3.0	12.0	60%	58.8	50.4	37.1	34.0	43.2	46.8
**25**	33.7	5.8	40.0	8.0	5.0	65%	63.4	61.9	50.3	41.1	57.5	72.0
**26a**	101.9	29.8	40.0	8.0	5.0	60%	70.7	69.4	54.6	29.6	66.6	72.0
**26b**	132.6	64.8	35.0	7.0	5.0	65%	55.5	54.7	51.6	38.4	53.8	59.5
**27**	71.6	17.1	40.0	8.0	5.0	60%	71.7	70.2	48.4	25.1	65.0	72.0
**28**	249.4	191.8	42.0*	3.0	14.0	60%	70.2	67.9	41.4	34.3	58.2	54.6
**29**	149.0	63.1	12.0*	4.0	3.0	60%	21.2	20.5	15.8	10.5	18.9	69.9
**30**	31.8	11.6	35.0	7.0	5.0	65%	53.9	52.1	43.8	35.7	46.9	59.5
**31**	78.8	27.8	40.0	8.0	5.0	65%	61.9	59.6	49.2	42.4	55.5	72.0

*ID 28: 35 Gy á 2.5 Gy with simultaneous integrated boost on the adrenal lesion to a cumulative dose of 42 Gy á 3 Gy

*ID 29: 45 Gy á 1.8 Gy with sequential boost on the adrenal lesion of 12 Gy á 4 Gy

*PTV* planning target volume, *GTV* gross tumor volume, *TD* total dose, *SD* single dose

## Results

The median age was 66 years (range: 27–82 years). Of all, 25 (25/31, 81%) patients suffered from advanced tumor disease with multiple metastases in different organs. 28 (28/31, 90%) patients showed an oligoprogression with a maximum of ≤3 metastases/tumor areas. In almost all patients (25/31, 81%), the primary tumor was controlled before irradiation. Isolated adrenal metastases without further tumor manifestation occurred in six (6/31, 19%) patients. Only two (2/31, 6%) patients reported symptoms related to the adrenal lesions, including back pain, flank pain, and abdominal pain; the applied RT had a positive effect on the pain situation in both patients. In 44% (15/34) adrenal lesions occurred synchronously during the diagnosis of the primary tumor, while 56% (19/34) of the metastases were diagnosed metachronously (> 3 months after initial diagnosis). The median time between the diagnosis of the primary tumor and the occurrence of adrenal metastases was 12.3 months (range: 0–169.0 months). Of all, 19 (19/31, 61%) patients received systemic tumor therapy, such as chemo, immuno- or hormone therapy, within four weeks before or after RT. It was ensured that this therapy was administered only in exceptional cases at the same time. The median planning target volume (PTV) was 76.8 ml (range: 24.1–434.8). Further planning and irradiation parameters are shown in Table 2.

### Outcome

**Fig. 1:**
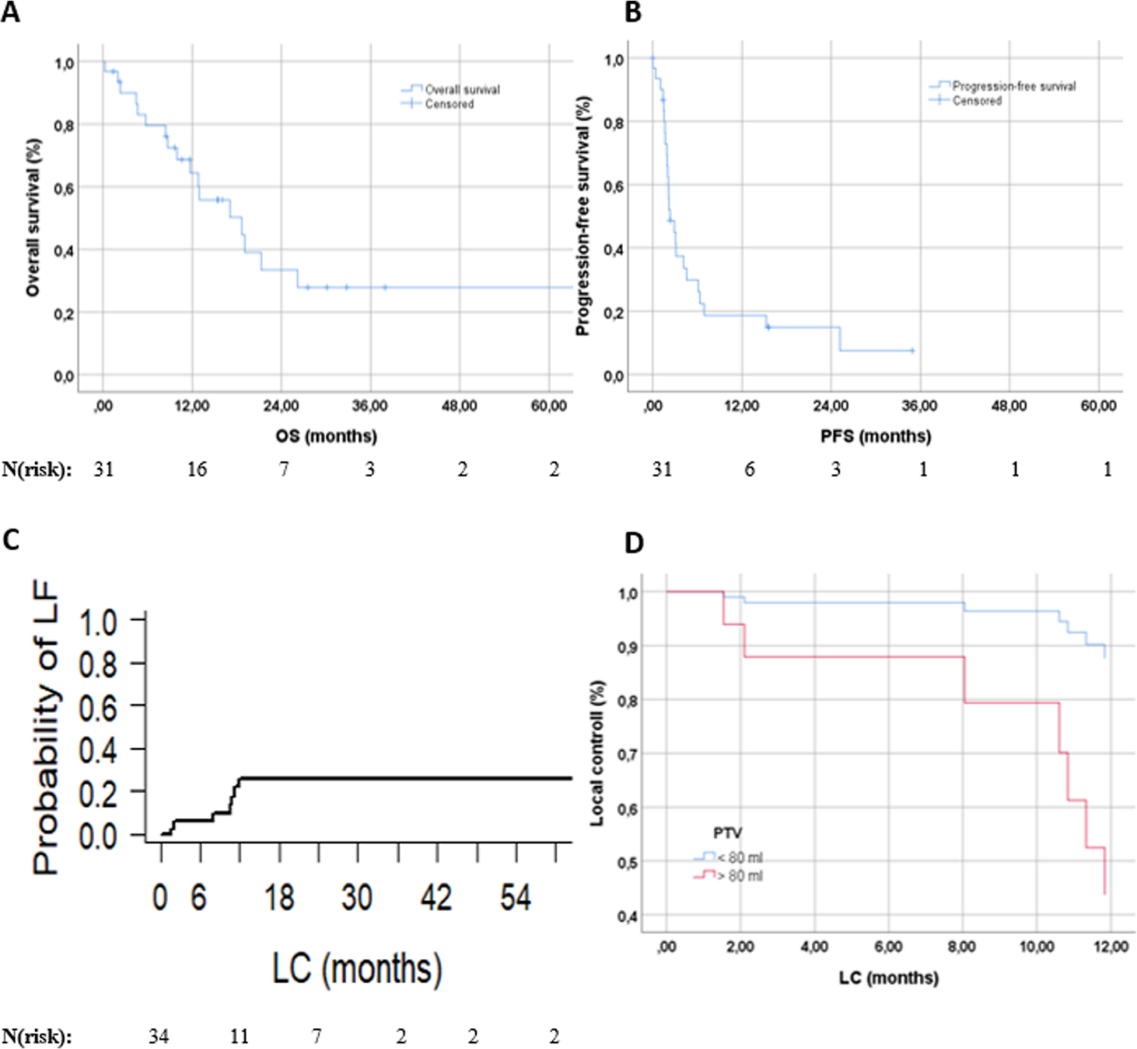
**a** OS of patients with AGMs treated with low-dose SBRT; **b** PFS of treated patients; **c** Probability of local failure; **d** LC divided into PTV </> 80 ml and adjusted for dose (*p* = 0.033)

Median follow-up for all patients was 9.8 months (range: 0–120.5), and 14.4 months (range: 1.4–120.5) for patients alive at the time of analysis. The mean LC was 79.0 months (95%-CI: 53.6–104.3; the median was not reached). We used competing-risk-analysis to estimate the probability of local relapse in our cohort. The 1-year chance of developing a local tumor progression was 25.9%, see also Table 3 and Fig. 1c. Median OS was 18.7 months (CI: 8.1–29.2; Fig. 1a), 64% of the patients were alive one year after RT. Median PFS was 2.3 months (CI: 1.0–3.6; Fig. 1b). After one year, 19% of the patients did not experience a distant failure (see also Table 3). We divided the patients into two groups according to PTV, with a threshold value of 80 ml and adjusted for dose using Cox regression (Fig. 1d). A PTV < 80 ml significantly influences LC (*p* = 0.033). For BED_10_, the ROC analysis could not identify a specific threshold; hence, we tested it as a continuous prognostic variable. No significant impact on LC (*p* = 0.115) could be found.

**Table 3:** LF, PFS, and OS in total and depending on time

			LF	PFS	OS
**Event (progress/death) - absolute/(%)**			7 (20.6)	25 (80.6)	17 (54.8)
**No event - absolute/(%)**			27 (79.4)	6 (19.4)	14 (45.2)
**Time (in months)**			mean: 79.0	median: 2.3	median: 18.7
**95%-CI (in months)**			53.6–104.3	1.0–3.6	8.1–29.2
	**Proportion surviving after**				
	**6-months**	**12-months**	**18-months**	**24-months**	
**OS**	80%	64%	50%	34%	
**PFS**	30%	19%	15%	15%	
	**Probability of LF after**				
	**6-months**	**12-months**	**18-months**	**24-months**	
**LF**	6%	26%	26%	26%	

*LF* local failure, *PFS* progression-free survival, *OS* overall survival, *CI* confidence interval

**Fig. 2:**
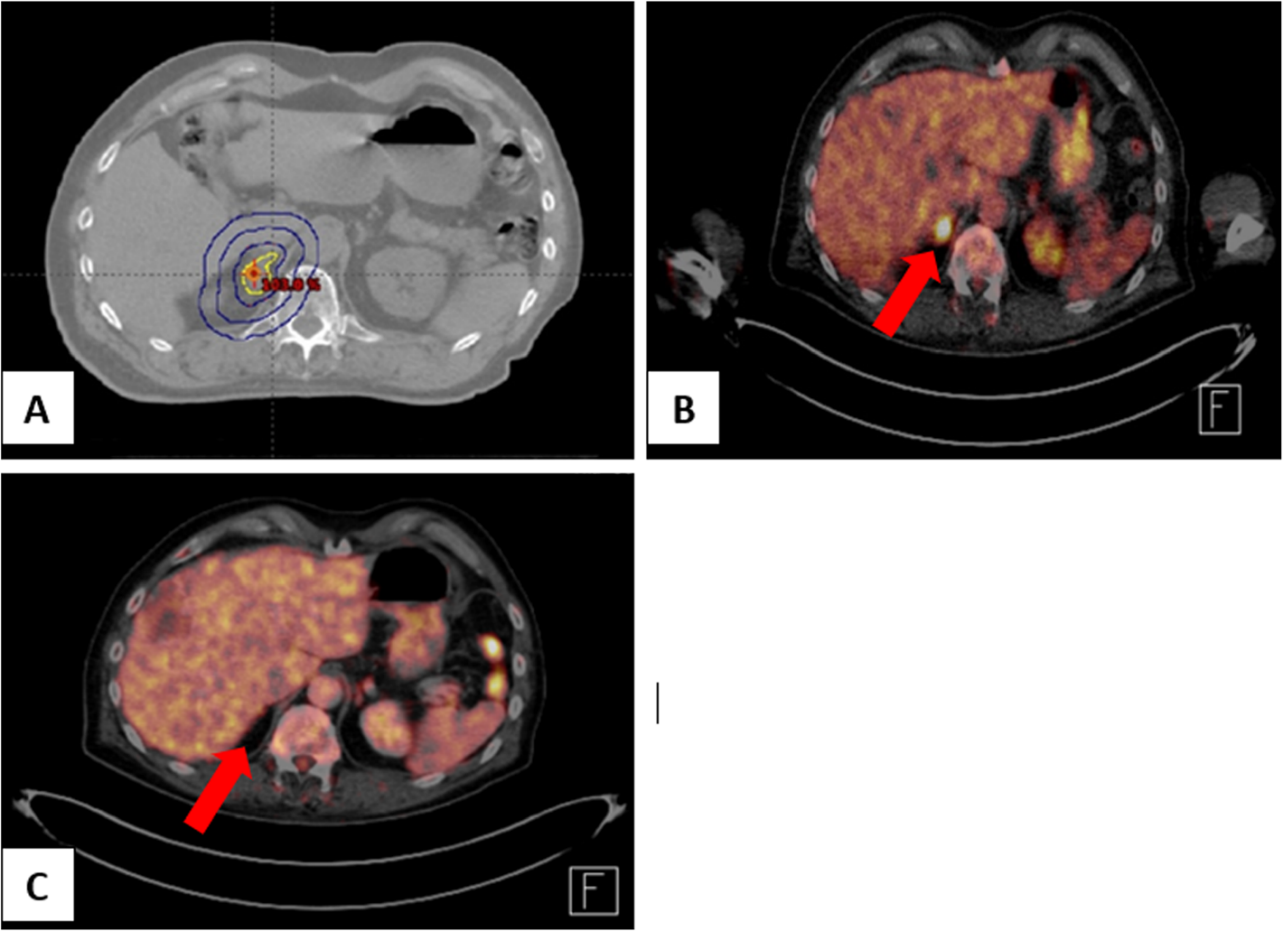
Example of a patient with malignant melanoma, who successfully underwent SBRT of adrenal gland metastasis. **a** CT scan and treatment plan before RT with 40 Gy in 5 fractions. **b** PET scan of the adrenal lesion before treatment (marked with red arrow). **c** PET scan four weeks after SBRT

Figure 2 shows an example case of a successfully SBRT-treated patient whos adrenal gland metastasis was no longer detectable after SBRT.

### Treatment toxicity

SBRT of the adrenal metastases was overall very well tolerated. No acute or late therapy-associated side-effects > grade 2 occurred. The most common side-effects included mild nausea, fatigue, loss of appetite, and abdominal pain, see Table 4. All acute symptoms were well treatable and were already decreasing after a short time. Two patients developed a mild adrenal insufficiency after SBRT and had to be treated with hormone substitution. Both patients were each irradiated with a cumulative dose of 35 Gy and 25 Gy with a single dose of 7 Gy and 5 Gy prescribed to the 65 and 60% isodose, respectively (ID 4 and 23, depicted in Table 2). No patient developed gastrointestinal ulcers, stopped SBRT early, or died during treatment.

**Table 4:** Acute and late toxicities after SBRT of adrenal gland metastases

**Acute toxicity (*n*** **= 31)**	**Grade 1 absolute/ (%)**	**Grade 2 absolute/ (%)**
**Nausea**	2 (6.5)	4 (12.9)
**Vomiting**	0 (0)	1 (3.2)
**Abdominal pain**	2 (6.5)	2 (6.5)
**Loss of weight**	1 (3.2)	1 (3.2)
**Loss of appetite**	2 (6.5)	1 (3.2)
**Diarrhea**	2 (6.5)	0 (0)
**Constipation**	1 (3.2)	1 (3.2)
**Fatigue**	6 (19.4)	5 (16.1)
**Throbbing pain**	0 (0)	2 (6.5)
**Adrenal insufficiency**	0 (0)	2 (6.5)
**Radiogenic gastritis**	0 (0)	1 (3.2)
**Flatulence**	1 (3.2)	1 (3.2)
**Late toxicity (*n*** **= 31)**	**Grade 1 absolute/ (%)**	**Grade 2 absolute/ (%)**
**Gastrointestinal**	4 (12.9)	0 (0)
**Fatigue**	1 (3.2)	3 (9.7)
**Headache**	0 (0)	2 (6.5)
**Loss of weight**	0 (0)	1 (3.2)

## Discussion

Hypofractionated low-dose stereotactic body radiation therapy of adrenal metastases has become increasingly important in recent years. We analyzed a cohort of 31 patients with 34 irradiated adrenal lesions. Occurred side-effects were extremely rare, mild, and well treatable. Local control was promising and comparable to other studies [6, 13, 14, 22–25].

In the past, surgical resection of adrenal metastases (adrenalectomy) was the gold standard for patients with isolated adrenal gland metastases. Especially if the metastases were isolated, the open or laparoscopic adrenalectomy was and is the first choice, depending on the tumor size and patient condition. Certainly, the postoperative side-effects associated with this invasive treatment should not be underestimated. Since 1992, the laparoscopic approach has been increasingly used as the postoperative complications are significantly lower, and both the duration and intensity, as well as the hospital stay can be reduced [3, 26–28].

Other treatment options include ablative procedures, such as cryoablation or radiofrequency ablation. However, these ablative measures bear a higher risk of blood pressure derailment and intensive post-intervention care [29]. In contrast to these invasive methods, SBRT is a non-invasive alternative with an outstanding risk-benefit profile. Due to the comparatively gentle tumor tissue inactivation and the biological effects, there is no associated secretion of catecholamines during therapy; thus, a therapy-associated hypertensive crisis is avoided, and adrenal hormonal function can be preserved in most cases, as our results showed [30].

According to Toesca et al., who compared the glomerular filtration rate (GFR) before and after stereotactic treatment, kidney function is also little or not affected by SBRT. According to several studies conducted in the past, LC after SBRT is equaled to or even better than ablative procedures [13].

The studies investigating RT of adrenal gland metastases conducted in the last six years report a 1-year LC rate between 73 - 97% [6, 13, 14, 22–25]. Our 1-year probability of local failure was 25.9%. The effect of BED_10_ on local tumor control has already been investigated in several studies, and it was noted that a higher applied dose tends to be associated with a better local outcome. Ippolito et al. reported an LC-rate of > 70% if BED_10_ is > 60 Gy and > 90% with BED_10_ > 90 Gy. In the cohort of Chance et al. appeared no local relapses by applying a BED_10_ > 100 Gy and Rudra et al. noted that all occurred local failures in their cohort were treated with the lowest mean BED_10_ of 43.2 Gy [8, 14, 31]. In our patients, we could not find any significant influence of BED_10_ on local control. Only a non-significant trend towards better local control with higher BED could be identified (*p* = 0.115). All adrenal metastases that showed a local relapse after RT were treated with a BED_10_ of 37.5 and 59.5 Gy. No local failure occurred with BED_10_ > 59.5 Gy. In order to better assess the correlation between BED_10_ and local tumor control in adrenal metastases, a larger group of patients would be necessary.

Therapy-associated toxicity is an important limiting factor of any new therapy. SBRT proved to be beneficial in our study as no side-effects >grade 2 occurred. Also, in other studies on SBRT of adrenal metastases, no grade 3–5 toxicity was reported [8, 14, 24, 25, 32, 33], except for grade 3 diarrhea in the patient cohort of Zhao et al. [6]. Due to the high precision and exact application of radiation, both acute and late toxicities are rare and usually very mild. However, due to the respiratory dependence of adrenal tumor volume, dosimetry may often prove very difficult for the multiple adjacent organs at risk, such as the liver and bowel. Excellent and precise respiratory tumor motion management is crucial during the radiation treatment, and the dose must be reduced or adjusted to the circumstances, especially to avoid late toxicity [24].

Furthermore, according to Holy et al., treatment on an empty stomach has a positive effect on the described side-effects, as it ensures a better reproducibility of the position of the internal organs, and the intestinal movements can be minimized [32]. Another factor influencing the onset of late-toxicities is the tumor volume. The smaller the irradiated lesion, the less likely it is the appearance of late therapy-associated side-effects, as Zhao et al. found in their study of 2018 [6]. Overall, our LC and treatment-associated toxicity results are very similar to previous studies, supporting the assumption that SBRT is a valid treatment option for metastases of the adrenal gland.

Limitations of the study were the relatively small patient cohort, the short follow-up, and the retrospective design. The heterogeneously designed irradiation regimens were also among the limiting factors, as no consistent treatment guidelines regarding fractionation and dose exist.

## Conclusion

Low-dose SBRT is an excellent, effective, and safe method to treat metastases in the adrenal gland. Due to the exact application and the precise dosing of the radiation, all toxicities have been low and easy to treat. High-precision RT is a promising alternative for patients for whom surgical treatment is not possible due to poor general conditions or other treatment-limiting factors. Based on our results, which support existing studies, SBRT should be considered as a therapy option for adrenal metastases as an alternative to surgical or systemic treatment. Prospective studies are nevertheless necessary for the validation of these results.

## Data Availability

All datasets used to create and support the results and conclusions of this article can be found within the article.

## Abbreviations

SBRT: Stereotactic body radiation therapy
LC: Local control
LF: Local failure
PFS: Progression-free survival
OS: Overall survival
IGRT: Image-guided stereotactic radiotherapy
CBCT: Cone-beam CT
CT: Computed tomography
MRI: Magnet resonance imaging
PET-CT: Positron emission tomography-CT
BED: Biological equivalent dose
DEGRO: Deutsche Gesellschaft für Radioonkologie
AGM: Adrenal gland metastasis
RT: Radiotherapy
NSCLC: Non-small-cell-lung-cancer
KPS: Karnofsky Performing Score
FU: Follow-up
CTCAE: Common Terminology Criteria Adverse Events
PTV: Planning target volume
GTV: Gross tumor volume
TD: Total dose
SD: Single dose
GFR: Glomerular filtration rate
CI: Confidence interval
ROC: Receiver operating characteristics
